# Correlation between Serum Steroid Hormones and Gut Microbiota in Patients with Alcohol-Associated Liver Disease

**DOI:** 10.3390/metabo12111107

**Published:** 2022-11-13

**Authors:** Bei Gao, Yixin Zhu, Weishou Shen, Peter Stärkel, Bernd Schnabl

**Affiliations:** 1 School of Marine Sciences, Nanjing University of Information Science and Technology, Nanjing 210044, China; wintergb@hotmail.com; 2 Key Laboratory of Hydrometeorological Disaster Mechanism and Warning of Ministry of Water Resources, Nanjing University of Information Science and Technology, Nanjing 210044, China; 3 Department of Medicine, University of California San Diego, La Jolla, San Diego, CA 92093, USA; y3zhu@ucsd.edu; 4 School of Environmental Science and Engineering, Nanjing University of Information Science and Technology, Nanjing 210044, China; wsshen@nuist.edu.cn; 5 Jiangsu Key Laboratory of Atmospheric Environment Monitoring and Pollution Control, Collaborative Innovation Center of Atmospheric Environment and Equipment Technology, Nanjing 211544, China; 6 Institute of Experimental and Clinical Research, Laboratory of Hepato-gastroenterology, Université Catholique de Louvain, 1200 Brussels, Belgium; peter.starkel@saintluc.uclouvain.be; 7 St. Luc University Hospital, Université Catholique de Louvain, 1200 Brussels, Belgium; 8 Department of Medicine, VA San Diego Healthcare System, San Diego, CA 92161, USA

**Keywords:** cortexolone, c-androsterone, subdoligranulum, ruminococcaceae, sporobacter

## Abstract

Alcohol-associated liver disease is a major public health concern globally. Alterations of steroid hormones and gut microbiota were both found in patients with alcohol-associated liver disease. However, their correlation has not been well characterized in these patients. In this study, we measured the level of 30 steroid hormones in serum and fecal samples collected from non-alcoholic controls, patients with alcohol use disorder, and patients with alcohol-associated hepatitis. The profile of serum and fecal steroid hormones was quite different in patients with alcohol-associated hepatitis from that in patients with alcohol use disorder and control subjects. Stronger alterations were observed in male patients than in females. Correlations were found not only between serum steroids and gut bacteria but also between serum steroids and gut fungi. These correlations need to be taken into consideration during the development of treatment strategies for alcohol-associated liver disease.

## 1. Introduction

Alcohol is a common addictive substance used for different reasons, such as entertainment purposes. Chronic and heavy alcohol consumption is associated with various diseases, including liver disease. Alcohol-associated liver disease is one of the most prevalent chronic liver diseases and a major cause of mortality globally. Alcohol-associated liver disease can range from mild forms, such as fatty liver, and moderate forms, such as steatohepatitis, to alcohol-associated hepatitis and cirrhosis. Patients with alcohol-associated liver diseases have abnormal levels of circulating steroid hormones [1]. Men with advanced liver disease are generally feminized and have more sexual dysfunction, with decreased testosterone and androstenedione and increased levels of estradiol and dehydroepiandrosterone sulfate levels [2].

Females usually are less likely to drink excessively than males. However, females who engage in problem drinking and alcohol use are more likely to develop alcohol-related medical problems [3]. Generally, females are more susceptible to alcohol than males, suggesting that sex and hormones might influence alcohol-associated liver injury [4]. In addition, gut microbiota plays an important role in the development of alcohol-associated liver diseases [5,6,7]. Sex is one factor that affects the gut microbiota [8]. The gut microbiota inhabit the digestive tract and encompass bacteria, fungi, archaea, and viruses. Alcohol-associated liver disease is not only associated with changes in gut bacteria but also in fungal populations [9].

The gut microbiota has effects on various biological processes, including the thyroid-gut axis and thyroid function [10]. The relationship between androgen and gut microbiota has been investigated in animal models. Androgen-driven gut microbiota shape the sexual dimorphism in glucose metabolism, as depletion of androgen altered the gut microbiota to be similar to females’ with improved glucose metabolism [11]. The interplay between estrogens and gut microbiota is bi-directional [12]. On the one hand, the gut microbiota could be regulated by estrogens. On the other hand, the gut microbiota harbors the genes known as “estrobolome”, which are capable of metabolizing estrogens [13]. Cholesterol is the building block of various hormones such as cortisol, aldosterone, estrogen, progesterone, and testosterone. Gut microbiota harbor the enzyme converting cholesterol to cholesterol sulfate, which affects the level of the building block for steroid hormones [14]. Bacterial products are known to affect the secretion of intestinal hormones, which further have an effect on metabolism in non-alcoholic fatty liver disease [15]. In our previous study, we found profound changes in the gut microbial composition, functional metagenome, and metabolome in patients with alcohol-associated hepatitis [16]. In addition, we have performed an integrative correlation analysis between gut microbiota and serum metabolome in patients with alcohol use disorder [17]. However, serum and fecal steroid hormones were not detected by untargeted metabolomics profiling in our previous studies. The interplay between hormones and gut microbiota remains to be determined in alcohol-associated liver disease.

In this study, we investigated the level of serum and fecal steroid hormones in both males and females with alcohol-associated liver disease and the correlation between serum steroid hormones and gut microbiota, including both bacteria and fungi. The findings from this study are helpful in improving the understanding of the correlation between hormones and gut microbiota in alcohol-associated liver disease and the development of gut microbiota-based therapeutic strategies.

## 2. Materials and Methods

### 2.1. Patient Cohort

This study includes 17 control subjects (14 males, 3 females), 32 patients with alcohol use disorder (25 males, 7 females), and 17 patients with alcohol-associated hepatitis (12 males, 5 females). Patients with alcohol-associated hepatitis were recruited from different sites, including Veterans Medical Center, San Diego, CA, USA; BCN Vall de’ Hebron, Barcelona, Spain; Yale University, New Haven, CT, USA; University of Wisconsin Madison, Madison, WI, USA; Monterrey, Mexico; Inserm, Lille, France; King’s College, London, UK; and Columbia University, New York, NY, USA. The inclusion criteria and exclusion criteria of patients have been described previously [16]. Patients with alcohol-associated hepatitis were not on steroids at the time of inclusion. Control subjects and patients with alcohol use disorder were recruited at the St. Luc University Hospital, Brussels, Belgium. Patients with alcohol use disorder met the criteria of the Diagnostic and Statistical Manual of Mental Disorders (Fourth Edition). The patients consumed more than 60 g of alcohol per day for more than one year. Control subjects were recruited with matched sex, age, and BMI and consumed less than 20 g of alcohol per day.

This protocol was approved by all participating medical centers. Written informed consent from each participant was obtained.

### 2.2. Extraction of Serum Steroid Hormones

Serum steroids were extracted from serum samples collected from 17 control subjects, 32 patients with alcohol use disorder, and 17 patients with alcohol use disorder. The extraction and quantification of steroid hormones have been described previously [18]. For serum samples, 50 μL serum samples were added to a polypropylene 96-well plate and extracted with 125 μL ACN:MeOH (1:1 *v*/*v*) solution. The serum samples were spiked with 25 μL 250 nM deuterated steroids as internal standards, 25 μL 0.2 mg/mL butylated hydroxytoluene and ethylenediaminetetraacetic acid as antioxidants, and 25 μL 1000 nM 1-cyclohexyluriedo-3-dodecanoic acid and 1-phenyl 3-hexadecanoic acid urea as quality markers. Then the samples were vortexed for 30 sec and centrifuged at 6 °C 15,000 rcf for 5 min. The supernatant was transferred to a 0.2 μm filter plate coated with polyvinylidene fluoride (PVDC) membrane and centrifuged at 15,000 g for 6 min. The solutions were collected and stored at −20 °C until analysis.

### 2.3. Extraction of Fecal Steroid Hormones

Fecal steroids were extracted from fecal samples collected from 17 control subjects, 29 patients with alcohol use disorder, and 17 patients with alcohol-associated hepatitis. For fecal samples, 1 mg of stool from each patient was used for extraction. A total of 10 μL antioxidant solution and 10 μL deuterated internal standards were added. Fecal samples were extracted with 500 μL cold methanol. Stainless steel grinding balls were added, and the samples were homogenized with GenoGrinder. The samples were centrifuged, and the supernatant was transferred to a 1.5 mL Eppendorf tube containing 10 µL 20% glycerol solution. The supernatant was extracted with 500 μL cold methanol and homogenized again. The supernatant was transferred to Speed-vac and evaporated to dryness. Samples were reconstituted in 100 μL ACN:MeOH (1:1 *v*/*v*) solution, which contains 100 nM 1-cyclohexyluriedo-3-dodecanoic acid and 1-phenyl 3-hexadecanoic acid urea as quality markers. The samples were vortexed for 10 sec and then sonicated for 5 min. Samples were then kept on ice for 15 min and centrifuged at 12,000× *g* for 3 min. The supernatant was transferred to a PVDC filter plate and centrifuged at 12,000× *g* for 6 min. The filtrate was collected and stored at −20 °C until analysis.

### 2.4. LC-MS/MS Analysis

Extracts were analyzed using liquid chromatography (Waters ACQUITY UPLC I-Class system, Milford, MA, USA). Water with 0.1% formic acid was used as mobile phase A; ACN with 0.1% formic acid was used as mobile phase B. The gradient is as follows: 0–0.5 min 10% mobile phase B, 0.5–1 min 10–20% mobile phase B, 1–1.5 min 20–22.5% mobile phase B, 1.5–11 min 22.5–45% mobile phase B, 11–12.5 min 45–95% mobile phase B, 12.5–16 min 95% mobile phase B, 16–16.5 min 95–10% mobile phase B, 16.5–20 min 10% mobile phase B. Acquity BEH C18 column (1.7 μm, 2.1 × 100 mm) was used for the separation of extracts, with a Vanguard precolumn. The column was maintained at 45 °C with a flow rate of 0.4 mL/min. The column was coupled to a Sciex 6500+ QTRAP hybrid, triple quadrupole linear ion trap mass spectrometer. The injection volume was 5 μL for each sample. Multiple reaction monitoring (MRM) was performed in positive ionization mode. Steroid hormones were quantified against 6-point calibration curves using internal standards. Analyst 1.6.3 was used for data acquisition. MultiQuant version 3.0.2 was used for the peak integration, peak area computation, and molar concentrations computation.

### 2.5. 16S rRNA Sequencing

The V4 region of the 16S rRNA gene was sequenced using the Illumina MiSeq V2 kit. DNA extraction, primers, PCR conditions, and data processing were described in our previous study [6,19]. Bacterial sequencing data can be found in the National Center for Biotechnology Information (NCBI) under BioProject PRJNA525701.

### 2.6. ITS Sequencing

Internal transcribed spacer 1 (ITS1) region was sequenced using Illumina MiSeq V2 kit. DNA extraction, primers, PCR conditions, and data processing were described in our previous study [19,20]. Fungal sequencing data can be found in the National Center for Biotechnology Information (NCBI) under BioProject PRJNA517994.

### 2.7. Statistical Analysis

MetaboAnalyst 5.0 was used to generate a PCA plot and heatmap [21]. Wilcoxon test was used for the comparison between the two groups. The false discovery rate (FDR) was calculated for the adjustment of *p*-values. MixOmics was used for the integrative and correlation analysis of serum steroids and gut microbiota [22]. Due to the sample availability, correlation analysis between bacterial genera and steroids was conducted in 3 control subjects, 29 patients with alcohol use disorder, and 17 patients with alcohol-associated hepatitis. Correlation analysis between fungal genera and steroids was conducted in two control subjects, three patients with alcohol use disorder, and 13 patients with alcohol-associated hepatitis. FDR less than 0.05 was considered significant in this study.

## 3. Results

### 3.1. Changes in Serum Steroid Hormones

**Figure 1:**
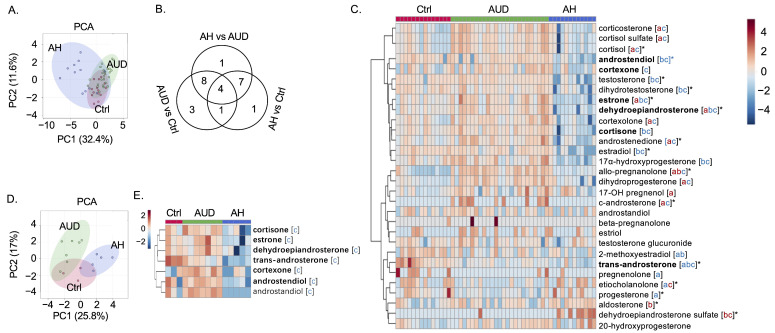
Serum steroid hormones in male and female patients. (**A**) Principal component analysis (PCA) of serum steroid hormones in male patients. (**B**) Venn diagram of significant (FDR < 0.05) serum steroid hormones in male patients. (**C**) Heatmap of 30 serum steroid hormones in male patients. * FDR < 0.05 in both serum and fecal samples; bold: FDR < 0.05 in both male and female patients. a: Patients with alcohol use disorder (AUD) vs. control subjects (Ctrl) FDR < 0.05, red: increase in patients with alcohol use disorder, blue: decrease in patients with alcohol use disorder; b: Patients with alcohol-associated hepatitis (AH) vs. control subjects FDR < 0.05, red: increase in patients with alcohol-associated hepatitis, blue: decrease in patients with alcohol-associated hepatitis; c: Patients with alcohol-associated hepatitis vs. patients with alcohol use disorder FDR < 0.05, red: increase in patients with alcohol-associated hepatitis, blue: decrease in patients with alcohol-associated hepatitis. (**D**) Principal component analysis of serum steroid hormones in female patients. (**E**) Heatmap of significant serum steroid hormones in female patients. c: Patients with alcohol-associated hepatitis vs. patients with alcohol use disorder FDR < 0.05, blue: decrease in patients with alcohol-associated hepatitis. Bold: FDR < 0.05 in both male and female patients.

Subjects’ characteristics are summarized in Table 1. Male patients with alcohol-associated hepatitis were separated from non-alcoholic controls and patients with alcohol use disorder in the PCA plot (Figure 1A). The number of significant steroid hormones is shown in Figure 1B when comparing the level of serum steroid hormones between different groups. Among 30 detected serum steroids, four were significantly different in three-way comparisons, including estrone, dehydroepiandrosterone, allo-pregnanolone, and trans-androsterone (Figure 1B). Hierarchical clustering of serum steroids showed that the profile of serum steroids in male patients with alcohol-associated hepatitis was quite different from that in male controls and male patients with alcohol use disorder (Figure 1C). Female patients with alcohol-associated hepatitis were well separated from female patients with alcohol use disorder in the PCA plot (Figure 1D). Seven serum steroid hormones were significantly reduced in female patients with alcohol-associated hepatitis compared with female patients with alcohol use disorder (Figure 1E). Notably, out of these seven serum steroid hormones, six were also significantly reduced in male patients with alcohol-associated hepatitis compared with male patients with alcohol use disorder (Figure 1C). The clinical parameters correlated with serum steroids in a sex-specific manner (Figures S1 and S2).

### 3.2. Changes in Fecal Steroid Hormones

**Figure 2:**
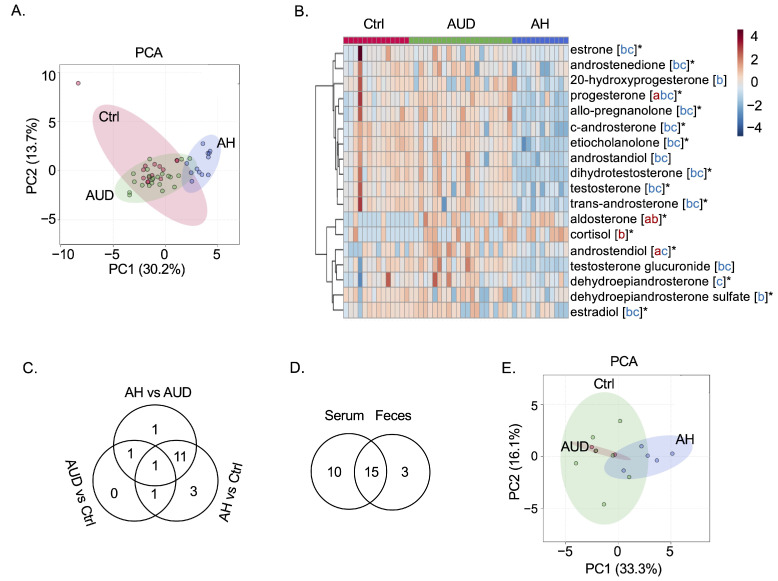
Fecal steroid hormones in male and female patients. (**A**) Principal component analysis (PCA) of fecal steroid hormones in male patients. (**B**) Heatmap of significant fecal steroid hormones in male patients. * FDR < 0.05 in both serum and fecal samples; a: Patients with alcohol use disorder (AUD) vs. control subjects (Ctrl) FDR < 0.05, red: increase in patients with alcohol use disorder; b: Patients with alcohol-associated hepatitis (AH) vs. control subjects FDR < 0.05, red: increase in patients with alcohol-associated hepatitis, blue: decrease in patients with alcohol-associated hepatitis; c: Patients with alcohol-associated hepatitis vs. patients with alcohol use disorder FDR < 0.05, blue: decrease in patients with alcohol-associated hepatitis. (**C**) Venn diagram of significant (FDR < 0.05) fecal steroid hormones in male patients. (**D**) Venn diagram of significant (FDR < 0.05) steroid hormones in both serum and fecal samples. (**E**) Principal component analysis of fecal steroid hormones in female patients.

Similar to serum steroid hormones, the profile of fecal steroid hormones in male patients with alcohol-associated hepatitis was separated from the two other groups (Figure 2A). A total of 18 fecal steroid hormones in males were significantly different, as shown in the heatmap (Figure 2B), among which progesterone was different in a three-way comparison (Figure 2C). A total of 15 steroid hormones were different in both serum and fecal samples in male patients (Figure 2D). The principal component analysis of fecal steroid hormones in females is shown in Figure 2E. No significantly different fecal steroids were found between female patients with alcohol use disorder and controls, between female patients with alcohol-associated hepatitis and controls, or between patients with alcohol-associated hepatitis and alcohol use disorder.

### 3.3. Correlation between Serum Steroids and Bacteria

**Figure 3:**
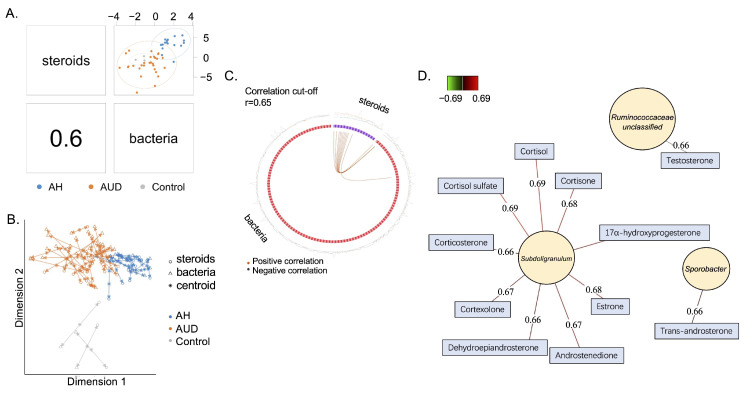
Correlated serum steroids and gut bacteria. (**A**) The overall correlation between serum steroids and gut bacteria is 0.6. (**B**) Integrative analysis between serum steroids and gut bacteria. Each sample corresponds to one arrow, with short arrows indicating strong agreement between two data sets and long arrows indicating a disagreement. (**C**) Correlations between serum steroids and gut bacteria with a cut-off set to 0.65. Orange line: positive correlation. (**D**) Correlations between serum steroids and gut bacteria. Ctrl, control subjects, AUD, patients with alcohol use disorder; AH, alcohol-associated hepatitis.

The overall correlation between serum steroid hormones and bacterial genera was 0.6 (Figure 3A). The integrative analysis between serum steroids and fecal bacteria is shown in Figure 3B. As shown in the circos plot, a positive correlation was found between 11 steroids and 3 fecal bacteria (Figure 3C). Specifically, *Sporobacter* was positively correlated with trans-androsterone; meanwhile, unclassified bacteria belonging to *Ruminococcaceae* were positively correlated with testosterone (Figure 3D). Interestingly, *Subdoligranulum* was positively correlated with nine serum steroids (Figure 3D). The correlation analysis between serum steroid hormones and bacterial genera in male and female patients is shown in Figure S3A,B (Tables S1–S3), specifically.

### 3.4. Correlation between Serum Steroids and Fungi

**Figure 4:**
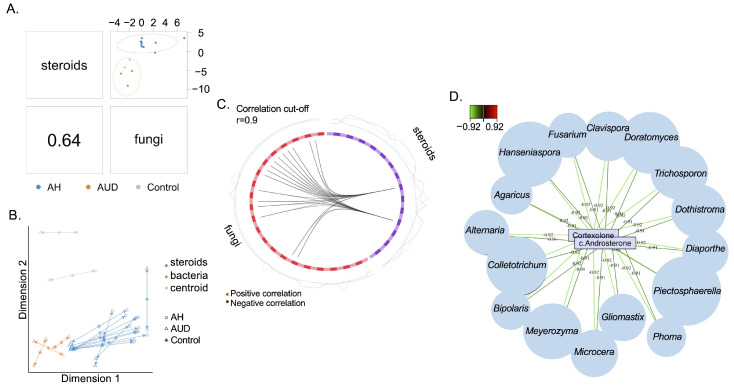
Correlated serum steroids and gut fungi. (**A**) The overall correlation between serum steroids and gut fungi is 0.64. (**B**) Integrative analysis between serum steroids and gut fungi. Each sample corresponds to one arrow, with short arrows indicating strong agreement between two data sets and long arrows indicating a disagreement. (**C**) Correlations between serum steroids and gut fungi with a cut-off set to 0.9. Black line: negative correlation. (**D**) Correlations between serum steroids and gut fungi. Ctrl, control subjects, AUD, patients with alcohol use disorder; AH, alcohol-associated hepatitis.

The overall correlation between serum steroid hormones and fungal genera was 0.64 (Figure 4A). The integrative analysis between serum steroids and fecal fungi is shown in Figure 4B. As shown in the circos plot, a strong negative correlation was found between two serum steroids and 16 fecal fungi (Figure 4C). Two serum steroids were cortexolone and c-androsterone, and the correlated fungal genera are shown in Figure 4D. The correlation analysis between serum steroid hormones and fungal genera in male patients is shown in Figure S4A (Tables S4 and S5).

## 4. Discussion

In this study, we found sex-specific changes in steroid hormones in patients with alcohol-associated liver disease, with stronger alterations observed in male patients than in females. Additionally, more alterations were found in serum samples than in fecal samples. Furthermore, we found various fecal steroid hormones not only correlating with gut bacteria but also with fecal fungi. This correlation needs to be taken into consideration in clinical practice when developing treatment strategies or recommending therapies for alcohol-associated liver diseases.

Cortisol is a stress hormone released by the adrenal glands. In this study, serum cortisol was increased in male patients with alcohol use disorder compared with controls and decreased in male patients with alcohol-associated hepatitis compared with patients with alcohol use disorder; meanwhile, fecal cortisol was increased in male patients with alcohol-associated hepatitis compared with patients with alcohol use disorder. Consistent with our study, the serum level of cortisol was increased in patients with alcohol use disorder [23,24]. Adrenal insufficiency was found in patients with severe liver disease, such as cirrhosis, recent liver transplantation, acute liver failure, and acute on chronic liver failure [25]. Insulin resistance is related to prolonged exposure to elevated levels of cortisol [26]. Changes in cortisol metabolism were also observed in patients with non-alcoholic fatty liver disease [27]. In a study population of infants at the age of 2.5 months, the diversity of gut microbiota is related to the stress response of saliva cortisol [28]. In the present study, we found cortisol was positively correlated with *Subdoligranulum*. *Subdoligranulum* is a strictly anaerobic Gram-negative bacterium, which is beneficial to patients suffering from necrotizing enterocolitis [29]. *Subdoligranulum* has been reported to be positively correlated with high-density lipoprotein cholesterol and microbial richness [30].

In the present study, we found a decrease in serum testosterone, dehydroepiandrosterone, and androstenedione in male patients with alcohol-associated hepatitis compared with patients with alcohol use disorder. Consistently, a reduction in testosterone was found in male patients with alcohol-associated cirrhosis [31]. Plasma testosterone and dehydroepiandrosterone increased after liver transplantation in male patients with alcohol-associated liver disease [32]. Serum testosterone was reduced in most male patients with cirrhosis, the level of which fell when the liver disease progressed [33]. Decreased dehydroepiandrosterone in plasma was found in patients with alcohol-associated liver cirrhosis [34]. Decreased testosterone, dehydroepiandrosterone, and androstenedione were also found in patients with non-alcohol-associated cirrhosis compared with control subjects [35]. Serum testosterone was positively correlated with the unclassified genus belonging to the *Ruminococcaceae* family in our study. Members of *Ruminococcaceae* are butyrate producers [36]. Gut *Ruminococcaceae* at baseline have been reported to correlate with the risk of antibiotic-associated diarrhea [37]. Injections of probiotics decreased stress-induced corticosterone and behaviors related to anxiety and depression [38].

We found serum corticosterone was increased in male patients with alcohol use disorder compared with control subjects. Corticosterone is a corticosteroid hormone of 21 carbons produced in the cortex of the adrenal glands. Injection of ethanol increased plasma corticosterone in male mice [39]. Serum corticosterone was positively correlated with *Subdoligranulum* in our study. Intestinal-derived corticosterone biosynthesis has been reported to be regulated by the intestinal microbiota, which further modulates the blood pressure in hypertension induced by high salt [40]. The gut microbiota could affect the production of corticosterone in the small intestine of mice [41]. Fructo-oligosaccharides treatment lowered plasma corticosterone levels, remodeled the gut microbiota, and alleviated depression-like behaviors in a rat model of stress [42]. In an obesity mouse model induced by a high-fat diet, high-intensity interval training, probiotic supplementation, and their combination reduced serum levels of corticosterone and improved mice’s anxiety-like behavior [43].

Estradiol was reduced in male patients with alcohol-associated hepatitis compared with controls and patients with alcohol use disorder. Estradiol is considered an endogenous antioxidant. In male patients with steatosis, reduced production of estradiol or the lower response to estradiol may be partly responsible for the progression of liver injury [44]. In animal models, hepatic fibrosis was suppressed by estradiol [45]. Allopregnanolone has been used for the treatment of alcohol use disorder [46]. The plasma level of allopregnanolone was increased in male adolescent humans by alcohol intoxication [47]. After ethanol consumption, a higher concentration of brain allopregnanolone was found in male C57BL/6 mice but not in female mice [39].

This study has several limitations. The sample size of the cohort is relatively small since we included patients only who were not treated with steroids. The findings need to be validated in a larger and independent patient cohort. In addition, a patient cohort with alcohol-associated cirrhosis is required as an appropriate control group for patients with alcohol-associated hepatitis, who, in the majority, have underlying cirrhosis. Further studies using animal models are warranted to elucidate the mechanisms behind the correlations between steroids, gut bacteria, and fungi in alcohol-associated liver disease. Since there are differences in the gut microbiota between humans and animal models, the findings found in our study provide valuable information for patients with alcohol-associated liver disease.

## Figures and Tables

**Table 1:** Subject characteristics.

	Non-Alcoholic Controls	Patients with Alcohol Use Disorder	Patients with Alcohol-Associated Hepatitis
Clinical parameter			
Total n	17	32	17
Age, years, n = 66	39 (27–71)	41 (27–59)	53 (32–75)
Body mass index (BMI), kg/m^2^, n = 66	22 (19–29)	24 (18–31)	28 (16–37)
Sex (male), n (%), n = 66	14 (82)	25 (78)	12 (70)
Laboratory parameter			
Albumin (g/dL), n =4 2		4.7 (3.9–5.2)	2.4 (1.5–3.5)
ALT (U/L), n = 49		37 (11–184)	47 (26–106)
AST (U/L), n = 49		41 (15–283)	136 (56–290)
Total bilirubin (mg/dL), n = 46		0.5 (0.2–1.1)	11.9 (3.1–36.2)
GGT (U/L), n = 40		41 (4–952)	243 (70–2860)
Platelet counts (×10^9^/L), n = 4 5		223 (21–434)	120 (55–414)
Creatinine (mg/dL), n = 46		0.8 (0.5–1.2)	0.9 (0.4–2.0)
International normalized ratio, n = 45		1.7 (1.1–4.4)	0.9 (0.8–1.2)
Fibrosis stage, n = 9			
Stage 1–3			n = 3
Stage 4			n = 6
CAP (dB/m), n = 31CAP > 250 dB/m, n (%)		284 (148–381)23 (74%)	
Fibroscan (kpa)		5.6 (3.1–7.0)	
MELD, n = 16MELD > 21, n (%)			23 (12–33) 11 (68%)

Note: Values are presented as median and range in parentheses (). The number of patients for which the respective data was available is indicated in the first column. ALT, alanine aminotransferase; AST, aspartate aminotransferase; GGT, gamma-glutamyl-transferase; CAP, controlled attenuation parameter; MELD, model for end-stage liver disease.

## Data Availability

Bacterial sequencing data can be found in the National Center for Biotechnology Information (NCBI) under BioProject PRJNA525701. Fungal sequencing data can be found in the National Center for Biotechnology Information (NCBI) under BioProject PRJNA517994.

## Supplementary Materials

The following supporting information can be downloaded at: www.mdpi.com/xxx/s1, Figure S1: Correlation between clinical parameters and serum steroids in male patients; Figure S2: Correlation between clinical parameters and serum steroids in female patients; Figure S3: Correlation between fecal bacteria and serum steroids; Figure S4: Correlation between fecal fungi and serum steroids in male patients. Table S1: Bacteria detected in stool samples; Table S2: Correlation between fecal bacteria and serum steroids in male patients; Table S3: Correlation between fecal bacteria and serum steroids in female patients; Table S4: Fungal genera detected in stool sample; Table S5: Correlation between fecal fungi and serum steroids in male patients.
