# Supplementary material for: Correlation between Serum Steroid Hormones and Gut Microbiota in Patients with Alcohol-Associated Liver Disease
Source: Metabolites. 2022 Nov 13;12(11):0. doi: 10.3390/metabo12111107 (PMC9699110; doi:10.3390/metabo12111107)
Supplement: Supplementary file 1 [file metabolites-12-01107-s001.zip › Supplemental Figures.pdf]

Supplemental Figures

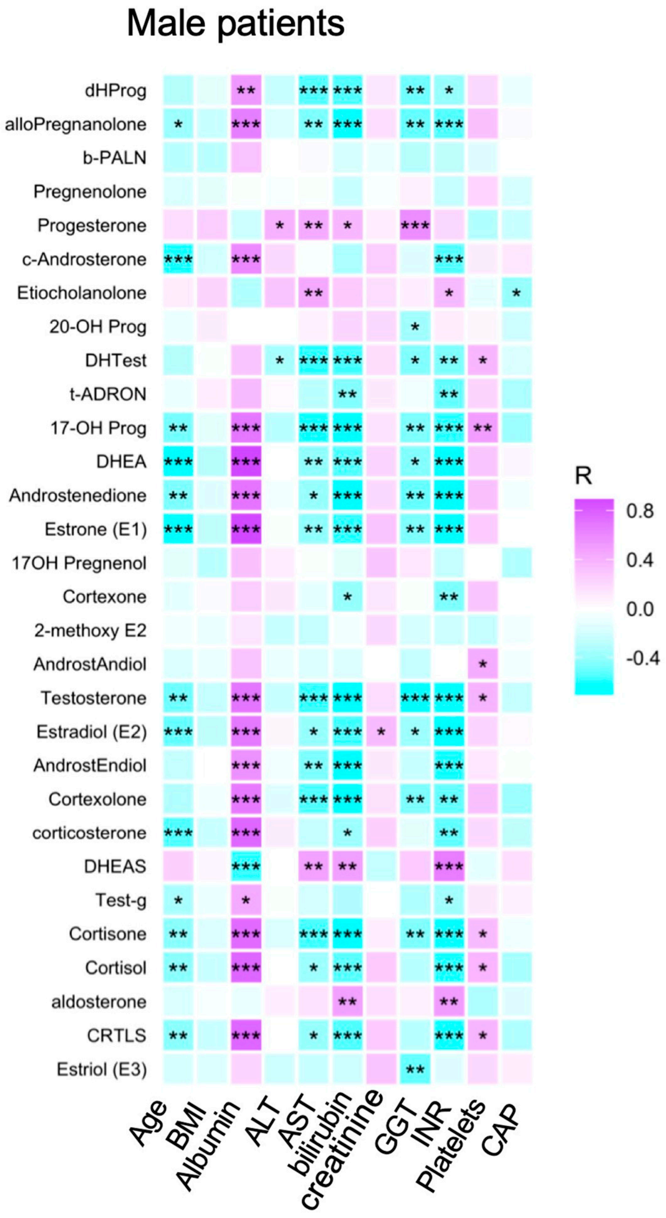

Figure S1. Correlation between clinical parameters and serum steroids in male patients. *: p<0.05, **: p<0.01, ***: p<0.001.

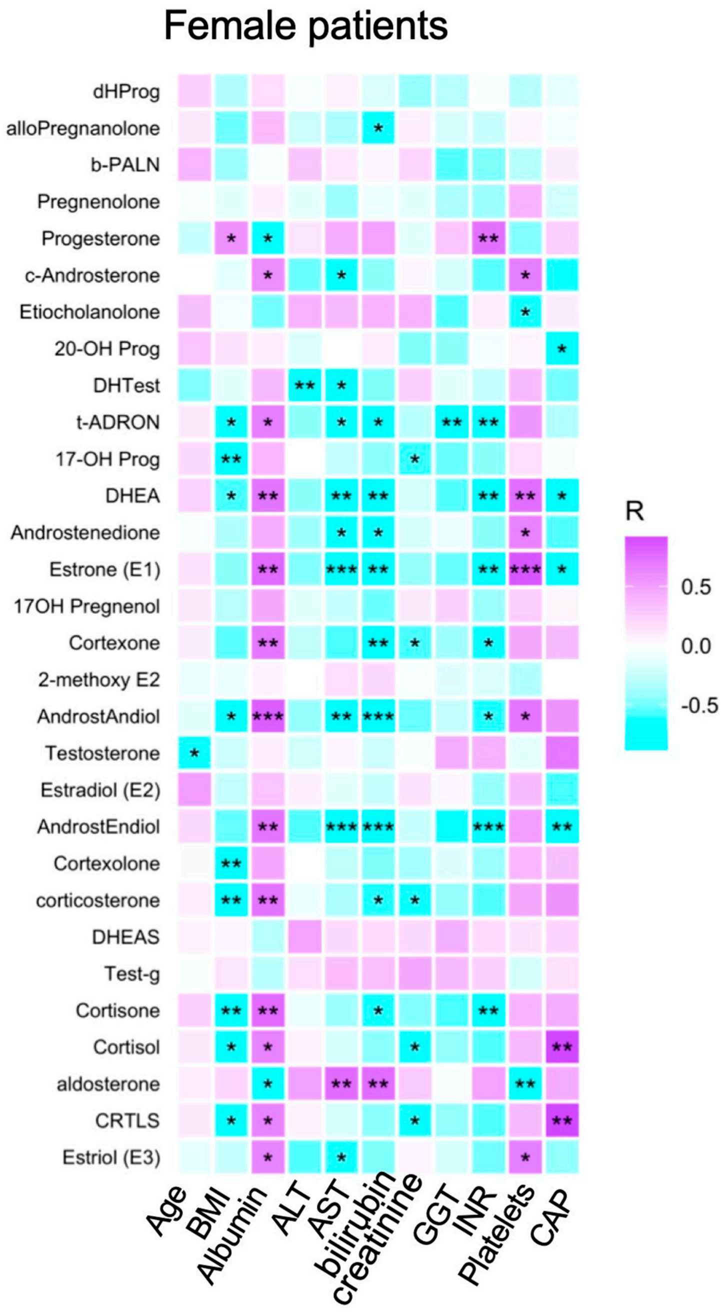

Figure S2. Correlation between clinical parameters and serum steroids in female patients. *: p<0.05, **: p<0.01, ***: p<0.001.

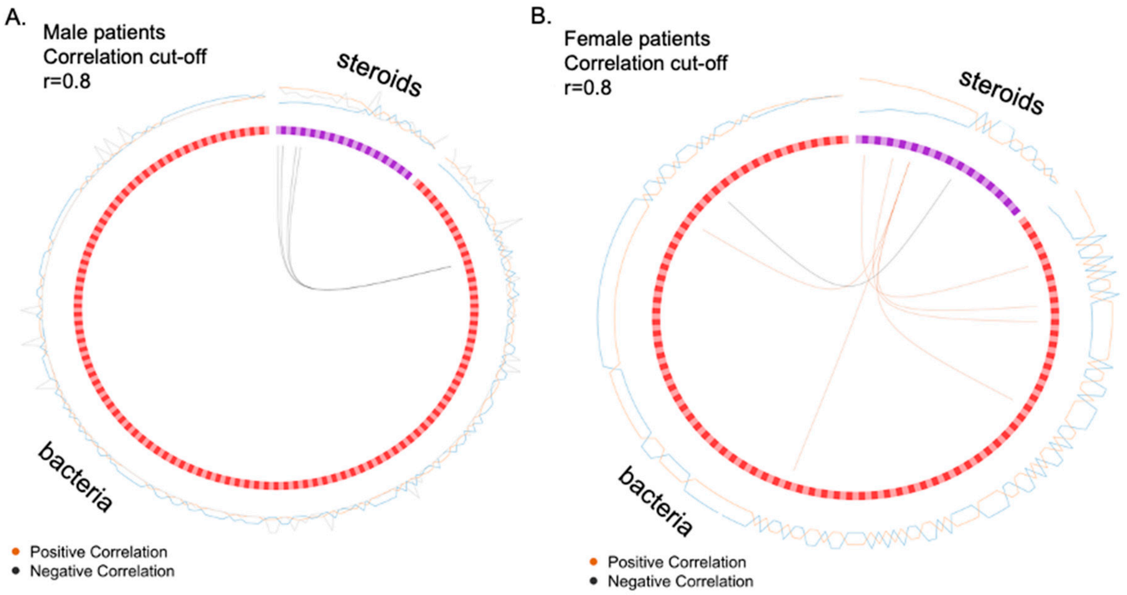

Figure S3. Correlation between fecal bacteria and serum steroids in male (A) and female patients (B). Correlation cut-off r=0.8.

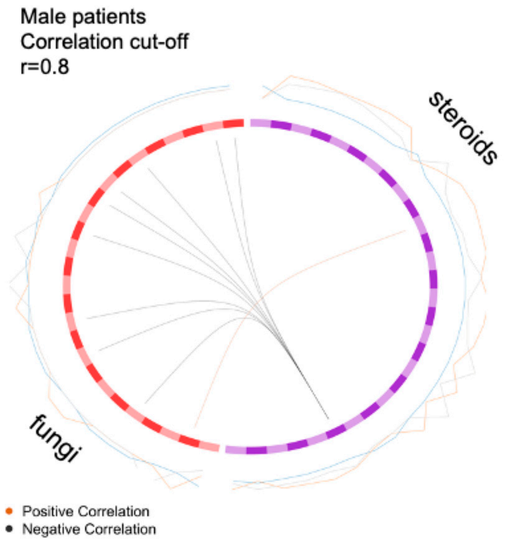

Figure S4. Correlation between fecal fungi and serum steroids in male patients. Correlation cut-off r=0.8.
